# Perspectives of Refugees from Ukraine on Cultural Identity and Health Care Experiences During U.S. Resettlement

**DOI:** 10.3390/nursrep15070263

**Published:** 2025-07-18

**Authors:** Marianne R. Choufani, Kim L. Larson, Marina Y. Prannik

**Affiliations:** 1 College of Nursing, East Carolina University, Greenville, NC 27834, USA; 2 Duke Healthcare System, Raleigh, NC 27609, USA; marina.prannik@duke.edu

**Keywords:** refugees, cultural identity, health care experiences

## Abstract

**Background:** More than three years have elapsed since the onset of the full-scale invasion of Ukraine by the Russian Federation, displacing millions of Ukrainians. While preserving cultural identity in the host country is important for gaining resilience among refugees, we found no studies about how cultural identity influences health care experiences during resettlement. **Objective**: This study explores how cultural identity shapes health care experiences among Ukrainian refugees during resettlement in the United States. **Methods**: We conducted an interpretive description study using focus groups to elicit the perspectives of Ukrainian refugees who resettled in North Carolina after 24 February 2022. Twelve Ukrainian women participated in one of four focus groups. Thematic content analysis was employed for case comparison, and themes were inductively derived. **Results**: Two themes were identified: troubled health care partnerships and imprecise notions of preventive practices. Troubled partnerships represented a lack of trust between refugees and U.S. clinicians and the health care system. Imprecise notions of preventive practices represented mistaken beliefs about prevention. **Conclusions**: This study adds to the science on refugee health in two ways. First, newly arrived refugees often maintain strong ties to their homeland, which shapes their health care decisions and reinforces their cultural identity. Second, despite being well educated, some refugees held misconceptions about preventive health care, highlighting the need for clinicians to provide clear guidance on primary and secondary prevention practices. Findings may help guide clinicians in delivering culturally sensitive care to refugee populations.

## 1. Introduction

The full-scale Russian invasion of Ukraine three years ago has forced millions of Ukrainians, mainly women and children, to leave their homeland and loved ones in search of safety. According to the United Nations High Commissioner for Refugees [UNHCR] [1], this is the largest refugee displacement in Europe since World War II. As of February 2022, there have been over 45 million border crossings out of Ukraine, and nearly 7 million Ukrainian refugees are currently recorded globally [1]. The war has caused the large-scale destruction of civilian lives and infrastructure since its onset [2]. By October 2024, adult and child casualties reached nearly 42,000, and the death toll reached nearly 13,000 [2]. Since the onset of the war, the United States has welcomed over 500,000 Ukrainian refugees through the Uniting for Ukraine (U4U) program and temporary visas [3]. However, following a recent presidential executive order, the United States Citizenship and Immigration Services (USCIS) has ceased accepting new U4U applications, creating uncertainty for Ukrainians currently in the United States under humanitarian parole [4].

Cultural identity is defined as “the fact of belonging to, or feeling that you belong to, a particular culture” [5] (para. 1). A sense of belonging and support in the host country has been shown to play a crucial role in the successful resettlement of Ukrainian refugees [6,7]. Those who relocated to Poland and Ireland often developed dual identities—one aligned with the host culture and another rooted in their identity as refugees [8]. In a 13-month longitudinal study with Syrian refugees in the Netherlands, fear of losing cultural identity was linked to persistent post-traumatic stress symptoms [9]. Similarly, a study involving Syrian refugees in Jordan and Turkey found that social media helped foster a sense of belonging [10]. To our knowledge, no studies have specifically explored how cultural identity influences the health care experiences of Ukrainian refugees in the United States.

### 1.1. Literature Review

This literature review includes studies published between 2022 and 2025. Search terms were broad to include emerging literature on refugees from Ukraine. The nascent literature has been organized to reflect the health concerns of refugees, as well as challenges and adaptations in resettlement.

#### 1.1.1. Health Concerns of Refugees

Women, children, and those with disabilities are the most vulnerable to war-related consequences [2]. Through a systematic review of the sexual and reproductive health needs of refugee women exposed to gender-based violence (GBV), Mathis and colleagues [11] emphasized the need for culturally relevant, trauma-informed care. A scoping review on the health and well-being of refugee women and girls calls for strategies that promote resilience and empowerment [12]. War conditions in Ukraine have exposed millions to emotional trauma, leading to adverse mental health outcomes. Among Ukrainian refugee women who gave birth in Lithuania, investigators reported high levels of postpartum depression [13]. Children are particularly vulnerable to the psychological effects of war. Many children have been displaced and/or separated from their families, further hindering access to health and social services [2]. Ukrainian children with previous pre-war adverse childhood experiences are at greater risk for mental health disorders [14], and they may lack the ability to cope with disasters, like war [15].

In addition to poor mental health outcomes, Ukraine has higher infectious disease rates and lower vaccination rates than other European countries [16]. Damage to the infrastructure in Ukraine has led to disruptions in chronic disease management [17,18], resulting in higher morbidity and mortality rates for refugees [19,20].

#### 1.1.2. Challenges and Adaptations in Resettlement

Refugees from Ukraine carry with them the direct effects of war into their host country resettlement process. Refugees who fled Ukraine for safety in other countries reported higher levels of anxiety and post-traumatic stress disorder (PTSD) compared to those who remained in Ukraine [21]. Studies from multiple European countries hosting refugees have found that post-migration difficulties, such as lost employment, diminished social status, and untenable housing, were associated with increased PTSD [22,23,24,25]. Numerous investigators report that language and communication differences among refugees and their host countries complicate access to health and social services [26,27,28,29,30]. Problems with language and communication can hinder preventive health care practices for refugees. A study with Ukrainian refugee mothers in Poland found communication barriers led to limited vaccination of children due to not knowing where, when, and what vaccines were necessary [31]. In another study, vaccine hesitancy among Ukrainian refugees was attributed to differences in vaccine schedules between Poland and Ukraine [32]. Refugee women from Ukraine expressed a lack of support for their infant feeding practices by health care workers in Poland, Israel, and the United States [33,34]. Challenges for Ukrainian refugees during resettlement have included an over-burdened health care system that is often costly and inaccessible [27,28,29,30]. The impact of refugee care on host country health systems has notably affected the workload of nurses, resulting in emotional trauma and burnout [32,35].

Refugee resettlement is a long-term process that requires navigating between two cultures. Refugees have gained personal resilience through a balance of maintaining their heritage and adapting to host country values and norms [36,37]. Resilience is defined as “the process and outcome of successfully adapting to difficult or challenging life experiences” [38] (para. 1). Several studies with Ukrainian refugees who resettled throughout the United States and Europe have found higher levels of resilience were associated with feeling culturally safe, a positive regard towards their cultural identity, and adaptation to a new environment [39,40,41,42].

In summary, refugees often carry the psychological trauma of war and violence into their host country. Resettlement is a continuous process that involves preserving, adapting, or redefining cultural identity while navigating between two cultures. Although maintaining cultural identity in the host country is crucial for building resilience, we found no existing studies that examine how cultural identity shapes health care experiences during resettlement. Therefore, this study aims to explore the influence of cultural identity on the health care experiences of Ukrainian refugees during resettlement.

### 1.2. Theoretical Concepts

This study was informed by Madeline Leininger’s Culture Care Theory [43]. Leininger’s Culture Care Theory provides a framework to understand how culture influences health. The theory is based on the notion that health beliefs and practices are shaped by culture, and as a result, nursing care should incorporate persons’ cultural identity in all aspects of care. Dr. Leininger, a nurse and anthropologist who created the Culture Care Theory, worked with more than a dozen cultures. This theory helps to inform caring based on people’s lifeways, time, and place, making it deeply rooted in the field of health anthropology [44]. Collaborative decisions between the individual/family and nurse are based on Leininger’s three modes of transcultural care actions and decisions: (1) culture care preservation, (2) culture care accommodation, and (3) culture care repatterning [43]. Preservation involves maintaining cultural beliefs and practices, accommodation involves adapting cultural practices for safe and effective care, and repatterning involves modifications of traditional practices for better health outcomes [45]. From our previous work, we were sensitized by concepts from two additional nursing models—Wikberg and Eriksson’s (2008) Intercultural Caring Model [46] and Campinha-Bacote’s Cultural Competency Model [47]. Concepts in both models support collaborative decision-making while incorporating micro- and macro-level factors.

## 2. Materials and Methods

### 2.1. Study Design

An interpretive description design using focus group discussions (FGDs) was conducted between October 2024 and March 2025. Interpretive description allows for a careful and systematic analysis of a phenomenon at both the descriptive (surface) and interpretive (deeper) levels, placed in the context of practice [48]. As a practice-based discipline, nursing aligns well with interpretive description, offering a valuable approach to exploring understudied phenomena—such as health care experiences in the context of refugee resettlement. Further, interpretive description enables the exploration of culturally rooted meanings in care experiences, making it well suited for this study.

The principal investigator (PI), a female nurse scientist with global health and school nursing experience, has worked with newly arrived Ukrainian children. Other members of the research team were three female bilingual (Ukrainian and English) Ukrainian health professionals who resided in the study region for over 10 years. These research team members were trained in research ethics and focus group methodology. They contributed their local knowledge of Ukrainian culture to this study. Throughout the study, the PI sought guidance from a female nurse scientist with expertise in qualitative methods and refugee and migrant health. The following research questions were addressed: (1) How does the cultural identity of refugees from Ukraine influence their health care experiences during resettlement? (2) How does the cultural identity of refugees from Ukraine influence collaborative nursing care during resettlement?

### 2.2. Ethical Considerations

The study was approved by the University and Medical Center Institutional Review Board (UMCIRB, ID# 24-001924). The PI was the only person with knowledge of participant names, which were separated from data and stored on a password-protected, encrypted university OneDrive. Each participant was assigned a unique identification number, and names and other identifiers were removed from the transcripts. The informed consent protocol included an explanation that participants did not have to answer all the questions, and they were free to leave the focus group at any time. The risk for participating in this research was minimal, although there was a possibility of re-traumatization when discussing sensitive topics. A list of local mental health support resources and national hotlines was available to participants if needed.

### 2.3. Study Setting

The PI met two Ukrainian nurses from the university where she had graduated. These nurses, along with other Ukrainian stakeholders, identified four communities in North Carolina (NC) as resettlement areas for refugees from Ukraine. Through these contacts, the PI attended Ukrainian-serving churches, met with Ukrainian advocacy groups, and attended Ukrainian festivals and an art exhibit in these communities. According to estimates by the NC State Refugee Office, since 2022, there have been approximately 3000–4000 Ukrainians who have resettled in NC; however, this is likely an underestimate.

### 2.4. Recruitment Strategy and Sample

**Figure 1:**
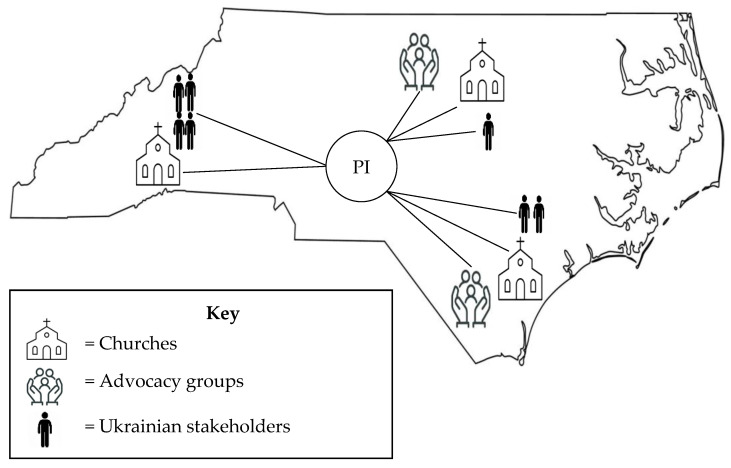
Social network of Ukrainians in NC.

The sample inclusion criteria were (a) resettled in the United States as a refugee as of Feb 24, 2022, (b) 18 years or older, (c) English or Ukrainian speaking, (d) identified as Ukrainian, and (e) having experience with the U.S. health and/or social service system. Exclusion criteria were being cognitively impaired and/or unable to sit for a 60 min focus group. We used snowball sampling that began with pre-established relationships with Ukrainian stakeholders living in NC. These stakeholders had emigrated from Ukraine to NC prior to February 2022, were respected members in their community, and had aided newly arrived refugees from Ukraine. Recruitment in each community was performed in person by the PI, who attended Ukrainian church services and other community events to describe the study, distribute flyers, and answer questions. Based on these connections, the PI established a social network of Ukrainians throughout the state of NC (see Figure 1).

The sample included 12 Ukrainian women who participated in one of four focus groups. Using information power, a sample size of 12–20 was determined appropriate, as the aim was narrow, the sample was specific, we used an established theory, and we conducted an exploratory analysis [49]. Focus group discussions (FGDs) were based on a protocol by Krueger and Casey [50] in which a minimum of 3 individuals/focus group is recommended. The PI proposed a date and time for the FGD and contacted each participant individually to confirm attendance on the proposed date/time. The PI contacted interested participants with reminders of the date, time, and location at three points: one week before, one day before, and the day of the FGD [50]. An agreement to participate in one FGD was received from a minimum of three Ukrainian refugees. However, at the scheduled time of FG2, only two participants were able to attend.

### 2.5. Data Collection

Theoretical constructs from Leininger’s Culture Care Theory [43], relevant literature, and a previous pilot study [51] informed the semi-structured interview guide used to elicit the perspectives of refugees from Ukraine. As moderator, the PI facilitated a conversational space with the intention of participants feeling free to share their perspectives and experiences. Ukrainian research team members served as co-moderators and interpreted as needed during FGDs for participants who chose to respond in Ukrainian. Both the moderator and co-moderator took field notes during the FGD and debriefed at the end of each session. For this study, we received a waiver of signed informed consent; still, all participants reviewed the consent document, checked a box indicating their agreement, and were given a copy of the document. A brief demographic form was completed by each participant. All forms and documents were available in English and Ukrainian.

All focus groups were conducted between December 2024 and January 2025 and ranged in length from 57 to 69 min. One FGD was conducted in person and three FGDs were conducted virtually. The location and format (in-person or virtual) of each FGD was determined by participant preference and residence. The in-person FGD was held in the classroom of a Ukrainian-serving church following the Sunday service. The moderator welcomed participants and reviewed informed consent and ground rules for focus groups, e.g., no right or wrong answers, and all opinions are valued. The co-moderator was present to assist with recording devices and take notes on relevant non-verbal cues. Virtual focus groups were conducted on a secure online platform (Teams^®^) (https://www.microsoft.com/en-us/microsoft-teams/group-chat-software, accessed on 30 May 2025). There are advantages (convenience, wider reach) and disadvantages (technology access and technical skills) to online focus groups [52]. We incorporated best practices for online focus groups, including a brief overview on using Teams^®^ technology before the sessions began, instructions with steps about how to participate, and descriptions and screenshots to troubleshoot problems [52]. Participants in the online groups kept their cameras on throughout the focus group. All participants received a $25 gift card as an incentive, and lunch and childcare were offered for the in-person focus group.

### 2.6. Data Management and Analysis

All focus groups were audio-recorded on two devices. The focus groups were audio-transcribed using Rev.com (https://www.rev.com/services/ai-transcription, accessed on 30 May 2025) and DeepL (https://www.deepl.com/en/products/translator, accessed on 30 May 2025) for language translation. English sections of transcripts were verified by the PI, and Ukrainian sections were verified by Ukrainian team members. To ensure translation accuracy, the three Ukrainian research team members cross-checked the transcripts with the original recordings for nuanced meanings or cultural references, then clarified and discussed these instances with the PI. This multi-step process, by a native speaker, preserved the integrity of the intended meaning and strengthened the study rigor. Transcripts were denaturalized, deidentified [53], and stored on OneDrive, a university-approved cloud storage system in compliance with university, state, and federal regulations.

The first cycle of coding began with reading the transcripts multiple times and creating memos [54] to document contextual factors and organize narratives. A codebook was created beginning with the first transcript and refined through an iterative process after each transcript to identify key words [53]. The codebook began with 35 code words, then it was merged into 15 coded segments. For example, treatment, health care, and practice were all merged as “care experience.” In vivo coding [53] was used to capture participants’ expressions, followed by the key word in context approach (KWIC) to condense code words into coded segments [54].

The early stages of analysis focused on what was said by participants at the surface level, while later analysis looked for trends or deviations, as well as what meanings could be applied to the participants’ words on a deeper level [48]. During each stage of analysis, we acknowledged biases, refined categories, and achieved consensus. Thematic content analysis was employed for case comparison and to structure the data so that a deeper, more interpretive understanding of their experiences could be made [48,55]. Themes were inductively derived using a variable-oriented strategy to determine similarities and differences between perspectives [53]. Primary variables were help, clinician, care experience, communication, and culture. Interpretations were guided by Leininger’s [43] theoretical constructs of preserving, accommodating, and repatterning cultural beliefs, and a cultural understanding of refugees from Ukraine was provided by Ukrainian stakeholders [51]. While we believe we reached code saturation based on guidelines from Hennink and Kaiser [56], we also recognize that in nursing, saturation may be less relevant, as the goal is to build on existing clinical phenomena, not to have come to an end point [57].

## 3. Results

Twelve Ukrainian refugees who resettled in NC following the Russian invasion of Ukraine on February 24, 2022, participated in this study. All participants were female and between 34 and 62 years of age, with an average age of 40. They left Ukraine between six weeks and nearly three years before the time of the study. All except two participants reported a religious affiliation, and language preferences included English, Ukrainian, and Russian. Most participants had post-secondary-level education (see Table 1).

The influence of cultural identity on health care experiences was identified in two themes: troubled health care partnerships and imprecise notions of preventive practices. Exemplars are used to demonstrate the categories and themes, with the focus group number presented after the exemplar.

### 3.1. Troubled Health Care Partnerships

The first theme, troubled health care partnerships, was represented by two sub-themes: relationships with U.S. clinicians and navigating the U.S. health care system. Participants described health care experiences as blending traditional practices with a new model of care in their host country. Negotiating between these two cultures often left participants confused and dissatisfied.

#### 3.1.1. Relationships with U.S. Clinicians

Some refugees continued communication with their Ukrainian physician, whom they described as their “family doctor” or their “main doctor.” Cultural identity was preserved through a trusted and long-term relationship with their doctor from Ukraine. This positioned refugees in between their trusted clinician and a newly established partnership with U.S. clinicians. Trouble was experienced particularly surrounding their freedom to choose their preferred plan of care, i.e., diagnostic tests and labs, and the U.S. clinician’s plan of care. In some instances, participants and their Ukrainian doctors were determined to find ways to maintain Ukrainian health care practices, even if that meant they “came up with a [false] story” (FG 4) to report to U.S. physicians:

For myself, I choose a doctor in Ukraine and it’s my doctor. Like main doctor, family doctor. And this is the person I trust for many years… and she says [Ukrainian doctor], you need to do the list of analysis and then when I go to doctor [United States], he called [and said] half of that list, you don’t need that. You just don’t need that. And I was like well I ask you to do this because my main doctor told me to do this. And I was refused, and it was not once, it was [multiple] times… She [Ukrainian doctor] recommended… You need to tell you have pain there and you have all the symptoms. I mean, it’s strange when you are going to doctor, and you need to lie to get all you need.

(FG 4)

This participant later shared that she was able to go to a private lab in the United States and request the analysis she wanted without a U.S. physician’s order by paying for the tests out of pocket. Similarly, she mentioned she also chose to pay for private health care in Ukraine, stating the quality and treatment of care is much better when receiving private vs. public care in Ukraine. Another participant described continued trust with her Ukrainian family doctor by contacting them for a second opinion on a treatment plan recommended by a U.S. physician. This ongoing communication with Ukrainian doctors made participants feel comfortable and at ease with their health care in the United States, especially when undergoing an unfamiliar procedure:

…it was unusual for us, when they said to remove wisdom teeth that had not yet grown. Well, it was the first time I’d ever seen that. To remove something that’s not there yet, and it’s like… no problem. We asked for an X-ray. And we consulted a doctor in Ukraine because I knew nothing about it, and the doctor said that there is such a practice and do it, and according to the x-ray, he also recommended it. And we had the operation, and we are happy with it.

(FG 1)

Troubled partnerships were observed when cultural identity was not respected in their U.S. health care encounters. One participant shared, “She [U.S. physician] didn’t like that my children weren’t vaccinated. Somehow it felt like we were beneath her” (FG 1). Alternatively, some participants noticed that U.S. physicians address the root cause of problems, a difference from their cultural practice in Ukraine.

While participants had fewer health care experiences with nurses than physicians, troubled partnerships were noted when nurses were “in the way” of their encounter with a U.S. physician. Participants described how nurses in the U.S. had a broader scope of practice compared to nurses in Ukraine. Yet, there were glimpses of refugee–nurse partnerships when a school nurse in the United States helped refugee parents with services to correct their child’s vision. While experiences with U.S. nurses were limited, cultural identity in nursing was discussed in one focus group in reference to family members in the nursing profession:

Participant 2: My mother used to be a nurse. Now she’s doing other stuff. So we practice evidential medicine. So, we don’t have any traditions or practices. Just pills, just doctors, just analysis.

(FG 4)

Participant 3: Yeah, and she [grandmother, a nurse] was a lover of like unconventional medicine, like taking herbs or something. And I was my grandma experiment. And she was healing me with all this, you know untraditional things and it was very good… So, I have a lot of touch with untraditional medicine and maybe that’s why I choose for my kids, homeopathic remedies.

(FG 4)

Although partnerships were troubled between refugees and clinicians in the United States, they did not eliminate the notion of collaboration. When asked how U.S. nurses and physicians could incorporate their cultural beliefs in health care, one participant described that seeking a basic understanding of their cultural practices would be beneficial: “There is already a situation that Ukrainians [refugees] are in America, so it would be worthwhile to listen, maybe, or at least to get acquainted with our protocols of treatment in order to just understand where we are coming from” (FG 1).

#### 3.1.2. Navigating the U.S. Health Care System

Troubled partnerships were also experienced with the U.S. health care system. Some refugees found it difficult to find providers who would accept Medicaid: “A big problem now for people who have this Medicaid is providers don’t want to work with Medicaid… And this is the most difficult to find a place who can provide you service” (FG 2). Others perceived difficulty with changing providers if they were unhappy with the care they received:

Because, of course, it’s easier to get to a doctor [in Ukraine]. I can choose a doctor. I have a choice… but here it’s so hard. You need to wait; you need to be covered by insurance. So, it’s a big difference… It’s easy to change doctors [in Ukraine].

(FG 2)

In addition, participants reported problems in understanding what services were covered by Medicaid. Participants shared that everyone in Ukraine had guaranteed health care through the National Health Service, a major difference between health care in Ukraine and the United States:

The program of medical guarantees in Ukraine actually provides you with guaranteed medical care for free which [the] government pays through the National Health Service. But the difference here [in the United States] [between] Medicaid and Medicare and [the] program of medical guarantees in Ukraine is the Medicaid is like about the level of income you have in family. In Ukraine, it doesn’t matter how much you make, you still qualify for health services, basic packages.

(FG 3)

Those refugees who were eligible for Medicaid and found a U.S. provider who accepted Medicaid expressed gratitude and regarded it as a privilege. Other participants who did not qualify for Medicaid found selecting a health insurance plan to be overwhelming, discouraging, and costly. Due to the challenges of navigating the U.S. health care system, some participants expressed a strong and cultural preference for the National Health Service in Ukraine:

I want to say the medicine in the United States is too expensive. But in Ukraine, we have the best… dentists, doctors, I am sure. I’m sure. I will do my teeth only in Ukraine. It’s a yes and in Ukraine we have free primary care. I can go to the doctor; I can choose doctor. I can take blood test. I can get a… Ultrasounds free and it’s a big difference. It’s amazing.

(FG 2)

Some participants required language assistance when navigating the U.S. health care system. Interpreters were perceived as inadequate or unavailable. Participants preferred having their children or friends interpret for them yet learned that this was a violation of health care policy. Some refugees’ experiences with interpreters were far more accessible in the United States than in other European countries, and they found their assistance to be extremely valuable.

Overall, participants experienced troubled partnerships with U.S. clinicians and the health care system. For some, this trouble was exacerbated by continued communication with their Ukrainian doctors and U.S. clinicians’ misunderstanding of Ukrainian culture. Language and cultural differences influenced their experience with the U.S. health care system.

### 3.2. Imprecise Notions of Preventive Practices

The second theme, imprecise notions of preventive practices, was characterized by two subthemes: a self-driven Ukrainian approach to prevention and a systems-driven U.S. approach to prevention. In some cases, there was clear rejection and confusion about prevention in both countries. Participants expressed strong cultural norms about prevention, often with mixed meanings. Some participants described preventive care in Ukraine as readily available and cost-effective. Others believed that prevention was not part of the Ukrainian culture but was paramount in the U.S. culture.

#### 3.2.1. A Self-Driven Ukrainian Approach to Prevention

Participants described prevention in Ukraine as self-driven because they got what they requested at the time they wanted it. Some participants experienced prevention in Ukraine as being able to select a clinician or specialist and request specific diagnostic tests. This cultural orientation to prevention was explained in this way:

So, I would say that preventive medicine is very popular in Ukraine. And if you want to see any doctor… You can just easily in that day or maybe the second day you can easily find the doctor and go get anything you want. If you want [an] MRI, you go and do [an] MRI and it won’t cost you a lot. I mean, in America, it will cost you probably like, I don’t know, $10,000.

(FG 4)

Self-driven approaches for participants included following their traditional Ukrainian diet of home-cooked, unprocessed foods and maintaining an active lifestyle:

People are used to cook[ing] home food. So it’s tradition to cook home food… We have a lot of walkways [in Ukraine] and you can easily get anywhere. You know when I’m walking in America, I feel like after zombie apocalypse because there’s no one [around].

(FG 4)

Alternatively, some participants denied the notion of prevention: “in Ukraine, we don’t have a culture to do any kind of screens, screenings, research and so on in advance” (FG 3). Some speculated that preventive care was not as culturally embedded in Ukraine because it was a less advanced health care system, with limited research and technology. Still, misunderstandings of preventive practices were evident, as noted by this participant:

When is it too bad, when it’s time to cut something off, they pay attention [in the United States]. In Ukraine, I go to the doctor and ask how I can prevent the development, and this is the most important thing for me.

(FG 2)

#### 3.2.2. A Systems-Driven U.S. Approach to Prevention

A systems-driven U.S. approach to prevention was described as one that forces a person to take better care of their health. For some, this came from a fear of getting sick and not being able to afford care, and for others, it came from a desire to achieve overall better health:

If they offer me to do some examinations according to my age, as a preventive measure, I agree to it, because I know that in America, I already understand that there is a system here, that they do everything to prevent all this. In Ukraine, they usually don’t do that. If there is a problem, [that is when] they go.

(FG 1)

Although some participants believed prevention was the focus of the U.S. health care system, they found it costly and inaccessible, as noted by this participant, “Unfortunately, in America I can’t afford prevention, that is, to have a full checkup once a year” (FG 3). Participants explained how preventive care in the United States required effort on their part to shift their practices to this change, such as scheduling care in advance, as opposed to waiting until a problem arises and then seeking care to prevent it from getting worse:

Of course, there are some advantages in American medicine, some advantages, we see that, but there is a lot that is not familiar to us. In Ukraine, we are used to getting to the doctor faster… Here it is a little more difficult, and we have to shift to this system, that it has to be done in advance, that it has to be done regularly, once a year, several times a year, to visit that doctor. We need to shift a little bit.

(FG 1)

In a systems-driven approach to prevention, access to resources was key. Participants emphasized how community programs provided essential assistance for prevention practices, especially local Ukrainian-serving churches. These community programs assisted with food, housing, household products, transportation, and spiritual needs. Additionally, community members assisted participants with translation in completing official documents for government benefits, health care, housing, and school registration:

When we came here, the people we lived with helped us. They helped us to register our children for school, to draw up documents, and the church helped a lot. There were also volunteer organizations all over the place (in the city they live in) that helped with food and the first necessary things that were needed for everyday life.

(FG 1)

Participants shared that the church was foundational in maintaining cultural identity for refugees through spiritual and social support: “It was just… Support for my faith. Then I found friends in the church. But then I got to know people. I started to socialize” (FG 2). One participant shared how her American and Ukrainian communities came together during the resettlement process:

And I lived with them [sponsors] during seven months here when I came. And [the] church that they are attending… they fundraised money to pay for my tickets [to] the USA. And then this organization they helped me to apply to all documents… Some of my [U.S.] friends they gave me a car… Ukrainian community helped me when I moved in this apartment. They helped me with some small things like plates, cups, some kitchen towels for first time.

(FG 3)

In general, there were mixed perceptions of prevention, making this term an imprecise concept for refugees from Ukraine. Some refugees were motivated by both a cultural self-driven approach and appreciated the U.S. systems-driven approach, while others misunderstood prevention altogether.

## 4. Discussion

We used interpretive description to better understand how cultural identity influences the health care experiences of Ukrainian refugees during resettlement. This study contributes to the science of refugee health in two ways. First, newly arrived refugees often maintain strong ties to their homeland, which shapes their health care decisions and reinforces their cultural identity. Clinicians should recognize this ongoing connection and incorporate it into their dialogue with refugees to foster collaborative care planning. Second, despite being well educated, some refugees held misconceptions about preventive health care, highlighting the need for clinicians to provide clear guidance on primary and secondary prevention practices. These findings offer a foundation for improving refugee–clinician collaboration by integrating cultural identity into the plan of care. Supporting cultural identity in care involves engaging refugees in meaningful discussions to understand their perspectives while addressing misconceptions and barriers to care.

The second aim of this study, to explore how cultural identity influences collaborative nursing actions with refugees from Ukraine, was only partially met, likely due to a less prominent role of nurses in Ukraine’s health care system [58]. Some noted that U.S. nurses have a broader scope of practice than their Ukrainian counterparts. The World Health Organization (WHO) [59] has initiated efforts to expand the role of nurses in Ukraine to address war-related health consequences. A glimpse of refugee–nurse collaboration emerged in interactions between school nurses and refugee parents. As school nurses are often the first point of contact for children’s health needs, they could play a pivotal role in leading refugee–nurse collaborations, particularly in promoting primary and secondary preventive care for children.

To interpret how cultural identity shapes health care experiences and nursing care during resettlement, we applied Leininger’s [43] three modes of transcultural care: preservation, accommodation, and repatterning. Culture care preservation was evident in refugees’ continued communication with Ukrainian physicians, allowing them to integrate familiar cultural practices into their U.S. health care experiences. Participants expressed trust with their Ukrainian family physician. Trust in the biomedical system constituted both a therapeutic and sociocultural relationship, further strengthening cultural identity. Personal relationships rooted in shared cultural expectations, familiarity, and freedom to make more decisions about their care likely led to a preference for Ukrainian health practices and health care providers. Most participants had been in NC for less than two years, limiting their time to establish similar relationships with U.S. providers. Some U.S. clinicians may have unintentionally acted on stereotypes—assuming refugees have lower education levels, socioeconomic status, or limited English proficiency. However, over 80% of participants in our study were well educated. Kohlenberger et al. [60] similarly found that highly educated Ukrainian refugees with strong language skills and higher socioeconomic status tended to resettle further west. In our study, higher education levels may have empowered participants to preserve their cultural identity. While they sought a partnership with U.S. providers, their expectations were often unmet. Some participants felt welcomed and motivated to engage in preventive practices while preserving their cultural identity, such as maintaining a traditional Ukrainian diet. This sense of welcome may stem from perceived cultural similarities between Ukrainian refugees and U.S. community members, church congregants, and friends. Research suggests that host communities may be more inclined to support Ukrainians due to perceived cultural proximity [7,28]. Participants emphasized the church’s role in sustaining cultural identity through spiritual and social support, which helped manage stress and potentially mitigated adverse mental health outcomes. Spiritual support and prayer have also been shown to foster resilience among Ukrainian refugees in Poland, Italy, and Spain [6]. Nurses and U.S. health care teams can support cultural preservation by approaching care with open-mindedness and a commitment to collaboration. This requires listening to and learning from refugees’ experiences, which shape their health care decisions and behaviors.

Culture care accommodation was reflected in refugees’ adaptation to the U.S. health care system. In Ukraine, care was often accessed on demand, whereas in the United States, scheduling and planning are essential. This shift led to dissatisfaction for some participants. Rolke et al. [61] also reported similar dissatisfaction among Ukrainian refugees in Germany regarding appointment scheduling and wait times. Clinicians can ease this transition by clearly explaining policies and procedures in refugees’ preferred languages. In Italy, for instance, Guidi et al. [40] found that both individualized and community-based interventions were necessary to help Ukrainian refugees transition from resilience to empowerment in their resettlement journey. Participants also had to adjust their communication strategies due to limited awareness of U.S. policies requiring qualified interpreters, which may have contributed to troubled partnerships. Many preferred family or friends as interpreters, believing they were more knowledgeable and accessible than agency interpreters. However, U.S. health care settings often mandate the use of certified interpreters or virtual language services like MARTTI (My Accessible Real-Time Trusted Interpreter). In contrast, refugees in Germany must often find and pay for their own interpreters [61]. Communication barriers were a recurring theme in our study and are well documented among Ukrainian refugees globally [27,28,29,30,31]. Nurses can help by ensuring access to qualified interpreters and explaining the rationale—such as privacy and accuracy—behind these policies. Understanding these requirements may lead to greater acceptance among refugees.

Culture care repatterning was observed as participants began to embrace the U.S. model of preventive care. Some expressed being open to annual checkups, recommended screenings, and making healthy lifestyle decisions, provided these services were affordable and accessible. However, navigating the U.S. health care system posed challenges. Participants found it more difficult to access preventive care in the United States than in Ukraine due to factors such as cost, confusion about where to go, and long referral and wait times. Similarly, Yeo and colleagues [26] interviewed stakeholders who worked in U.S. refugee resettlement and found that refugees from many different countries struggled to navigate the U.S. health care system, reporting preventive care as a major concern. In Ukraine, they were accustomed to receiving lab tests or screenings promptly upon request. Participants described the need to repattern their approach to care by planning and scheduling well in advance. These findings are congruent with those of Childress and colleagues [28], who also reported that Ukrainian refugees had to adjust their health care practices to align with the U.S. model. Similarly, in other high-income countries, such as Australia and the UK, refugees struggled with appointment-based, preventive health care services [62]. The cost of health insurance was the primary barrier for participants in this study to repatterning their preventive health practices. Having come from a country with a National Health Service orientation [63], participants were unaccustomed to paying for health care. This sample of young adult women, many with small children, would likely benefit from both primary and secondary preventive health services. Nurses and U.S. health care teams can engage with refugees to build a mutual understanding of preventive care in both the U.S. and Ukraine. Such partnerships could lead to collaborative decision-making and actions, potentially requiring repatterning, to enhance access to and the utilization of preventive services in the United States.

**Figure 2:**
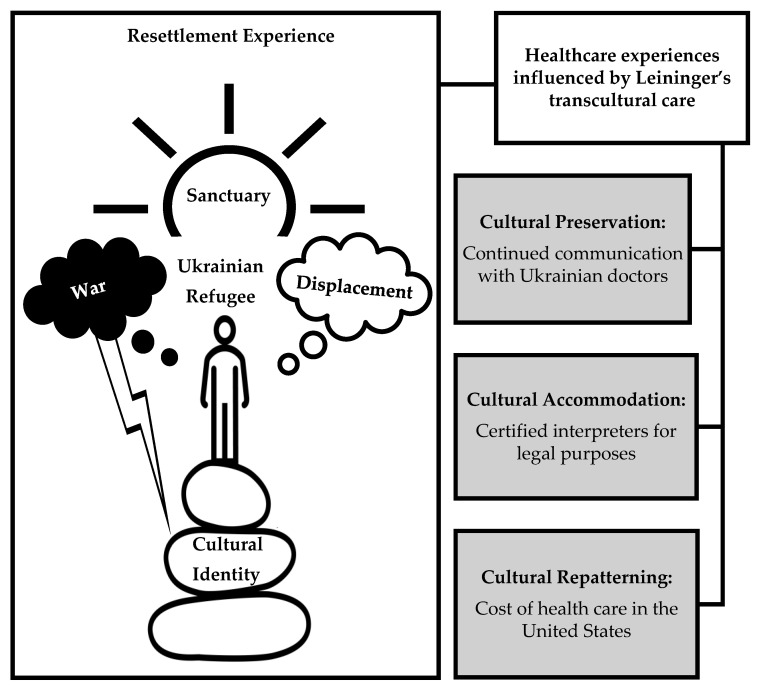
Cultural identity and health care experiences during resettlement.

This study followed a focused ethnography that we conducted with Ukrainian nurses who worked in NC and aided newly arrived Ukrainian refugees [51]. In that study, a conceptual model was generated from the perspectives of Ukrainian nurses that depicted refugee resettlement. This study builds on that model by adding the perspectives of refugees (Figure 2). On the left side of the figure, the cultural identity of Ukrainian refugees is symbolized by a stack of rocks, representing their foundation. War-related trauma is shown as a lightning bolt striking this foundation, threatening their cultural identity. Displacement is depicted by a cloud overhead, and the church symbolizes sanctuary and community support. The model now incorporates the voices of Ukrainian refugees in transcultural care: preservation, accommodation, and repatterning, and this deepens its cultural relevance.

### 4.1. Implications for Nursing Research, Practice, and Education

Cultural identity shapes health care experiences through culture care preservation, accommodation, and repatterning. However, the underlying factors that support or hinder these processes require further investigation. Future nursing research could explore which sociocultural factors affect refugees’ ability to integrate their cultural identity with U.S. health care practices. As Ukraine is classified as an upper-middle-income country [64], Ukrainian refugees left behind full lives, financial stability, and established careers. Research is needed to understand whether forced displacement from such backgrounds impacts their willingness to adopt new cultural practices in host countries. Further studies might also identify specific actions that nurses and other clinicians can take to improve refugees’ understanding, access, and use of preventive health care in the United States. Although most participants in this study were college-educated, many expressed confusion about the concept and availability of preventive health services in both Ukraine and the U.S. This suggests that solutions must go beyond educational interventions and focus on making preventive care accessible where refugees live, work, and learn.

The findings offer practical insights for nurses by promoting culturally sensitive care to help refugees build resilience. School nurses play a pivotal role in caring for refugee children and can lead efforts in parent–nurse collaboration. In this study, school nurses effectively met refugee families where they were—providing critical knowledge about where and how to access preventive care. Although some participants expressed hesitancy in collaborating with nurses, promising examples emerged in school settings. Nurses can help preserve cultural identity by advocating for bilingual education programs [65]. Nurses should become knowledgeable about the long-term implications of current policies affecting refugees. Current U.S. policies leave refugees without a pathway to permanent residency. Advocacy for more compassionate immigration solutions might include expanded immigration pathways and job training to prepare refugees for a potential return to their home country. For meaningful collaboration, however, nurses need adequate time, culturally relevant assessment tools, and system-wide support—including access to qualified interpreters. The Ukrainian stakeholders in this study, primarily nurses, informally supported fellow refugees outside their professional roles, aiding community members from churches, families, and even strangers. Their involvement highlights the importance of partnerships between nurses and informal helpers, which should be actively encouraged and supported [26]. While European countries have provided health insurance coverage to Ukrainian refugees through the EU’s Temporary Protection Directive [61], U.S. nurses and health care teams can advocate for Medicaid coverage for refugees, at least until other insurance options become available. Policymakers and clinicians can foster resilience and well-being by supporting refugees’ psychological recovery, social integrations, and long-term health outcomes [39].

Nursing education must reflect the need for a deeper understanding of refugee health and cultural identity. To collaborate effectively, nurses must first build cultural competence. Curriculum enhancements, including refugee-focused content and simulation scenarios, may better prepare nurses to serve refugee populations [32]. Modules on trauma-informed care are being included in nursing curricula [66,67]. Clarity around preventive care, especially vaccinations and screenings, is essential. Nurses should be trained to engage refugees in culturally meaningful conversations about prevention. Ukrainian nurses on this research team contributed cultural expertise and a deep commitment to supporting positive resettlement outcomes. This same motivation may also be present in nursing students passionate about refugee health. Encouraging openness to cultural nuances can foster collaborative, culturally sensitive care [68]. In alignment with the State of the World’s Nursing report, which calls for investment in nursing education, jobs, leadership, and service delivery [69], the U.S. could support Ukrainian nurses by recognizing their educational credentials or offering pathways to U.S. equivalents. Additionally, inviting Ukrainian nurses to speak at events such as International Nurses Day or the Sigma Nursing Honors Society program could amplify their contributions and promote cross-cultural learning.

### 4.2. Strengths and Limitations

The greatest strength of this study was the contribution by the Ukrainian research team members, well-respected members of their community, who informed the study design, data collection and analysis, and dissemination of findings. Research on the resettlement of refugees from Ukraine in the U.S. is limited. To our knowledge, no studies have been found with a focus on cultural identity and health care experiences of refugees from Ukraine in the United States during resettlement. Still, we acknowledge several limitations in this study. The sample was all female and well educated, which limits the transferability of findings. Greater diversity in gender, age, and educational level might yield broader insights. Additionally, only two participants attended FG2, which weakens the group dynamics typical of FGDs. Nonetheless, the sample included refugees from four different communities across the state with various resettlement experiences and perspectives. Research conducted in two languages is challenging, and while the PI did not speak the Ukrainian language, native speakers ensured translation accuracy throughout the research process. Lastly, the findings suggest that participants interacted more frequently with physicians than with nurses; therefore, the nursing-related insights should be considered exploratory at best.

## 5. Conclusions

This study underlines the complexity of preserving, accommodating, and repatterning the cultural identity of Ukrainian refugees within the U.S. health care system. Newly arrived refugees often maintain strong ties to their homeland, which shapes their health care decisions and reinforces their cultural identity. Despite being well educated, some refugees held misconceptions about preventive health care, highlighting the need for clinicians to provide clear guidance on primary and secondary prevention practices. Nurses can help preserve cultural identity by advocating for bilingual education programs and lobbying for system-wide support for curricular enhancements and professional development focused on trauma-informed care and refugee health. Ukrainian stakeholders proved to be important leaders in refugee resettlement communities. Their involvement should be supported and encouraged. Findings from this study can serve as a foundation for fostering refugee–nurse collaboration that supports the preservation of cultural identity, while promoting positive resettlement and health care experiences in the United States.

## Figures and Tables

**Table 1:** Participant demographics by focus group (FG).

Characteristic	FG1 (*n* = 3)	FG2 (*n* = 2)	FG3 (*n* = 4)	FG4 (*n* = 3)	Total (*N =* 12)
Gender					
Female	3	2	4	3	12
Age					
25–34	0	0	0	1	1
35–54	3	1	4	2	10
55–65	0	1	0	0	1
Time in United States					
Less than 1 year	1	0	2	0	3
1–2 years	2	2	2	2	8
>2 years	0	0	0	1	1
Preferred language					
Ukrainian	3	0	2	0	5
English	0	0	1	0	1
Russian	0	1	0	0	1
Mix: Ukr., Eng., Rus.	0	1	1	3	5
Marital status					
Married	3	0	3	1	7
Single	0	1	1	1	3
Divorced	0	1	0	1	2
Preferred religion					
Baptist/Protestant	3	0	1	1	5
Catholic	0	1	2	0	3
Other: Orthodox Christian	0	0	1	1	2
None	0	1	0	1	2
Highest educational level					
Finished 12th grade	1	0	0	0	1
Vocational training	1	0	0	0	1
College-level degree	1	2	4	3	10
Employment					
Full-time	2	1	1	2	6
Part-time	0	0	2	1	3
Unemployed	1	1	1	0	3

## Data Availability

The original contributions presented in this study are included in the article. Further inquiries can be directed to the corresponding authors.

## Public Involvement Statement

No public involvement in any aspect of this research.

## Guidelines and Standards Statement

This manuscript was drafted against the Consolidated Criteria for Reporting Qualitative Research (COREQ).

## Use of Artificial Intelligence

Rev.com (https://www.rev.com/services/ai-transcription, accessed on 30 May 2025) was used for audio transcription. English sections of transcripts were verified by the PI. During the preparation and implementation of this study, the authors used DeepL (https://www.deepl.com/en/products/translator, accessed on 30 May 2025) for the purposes of language translation. English study materials were translated to Ukrainian for participants. Portions of focus groups spoken in Ukrainian were translated to English for data analysis. Native Ukrainian speakers on the research team verified all translations from DeepL. Microsoft Copilot (https://copilot.microsoft.com/, accessed on 30 May 2025) assisted with grammar and structure in the manuscript revision. The authors have reviewed and edited the output and take full responsibility for the content of this publication.

## Abbreviations

The following abbreviations are used in this manuscript:

UNHCR	United Nations High Commissioner for Refugees
U.S.	United States
U4U	Uniting for Ukraine
USCIS	United States Citizenship and Immigration Services
GBV	Gender-based violence
PTSD	Post-traumatic stress disorder
FGD	Focus group discussions
PI	Principal investigator
UMCIRB	University and Medical Center Institutional Review Board
NC	North Carolina
KWIC	Key word in context approach
FG	Focus group
WHO	World Health Organization
MARTTI	My Accessible Real-Time Trusted Interpreter
